# Overcoming Limitations of Cisplatin Therapy by Additional Treatment With the HSP90 Inhibitor Onalespib

**DOI:** 10.3389/fonc.2020.532285

**Published:** 2020-09-30

**Authors:** Anja Charlotte Lundgren Mortensen, Tabassom Mohajershojai, Mehran Hariri, Marika Pettersson, Diana Spiegelberg

**Affiliations:** ^1^Department of Immunology, Genetics and Pathology, Uppsala University, Uppsala, Sweden; ^2^Department of Surgical Sciences, Uppsala University, Uppsala, Sweden

**Keywords:** cisplatin, Hsp90 inhibition, drug resistance, synergy, combination treatment, chemo-sensitization, AT13387, CDDP

## Abstract

**Rational:**

Cisplatin based cancer therapy is an affordable and effective standard therapy for several solid cancers, including lung, ovarian and head and neck cancers. However, the clinical use of cisplatin is routinely limited by the development of drug resistance and subsequent therapeutic failure. Therefore, methods of circumventing cisplatin resistance have the potential to increase therapeutic efficiency and dramatically increase overall survival. Cisplatin resistance can be mediated by alterations to the DNA damage response, where multiple components of the repair machinery have been described to be client proteins of HSP90. In the present study, we have investigated whether therapy with the novel HSP90 inhibitor onalespib can potentiate the efficacy of cisplatin and potentially reverse cisplatin resistance in ovarian and head and neck cancer cells.

**Methods:**

Cell viability, cancer cell proliferation and migration capacity were evaluated *in vitro* on models of ovarian and head and neck cancer cells. Western blotting was used to assess the downregulation of HSP90 client proteins and alterations in downstream signaling proteins after exposure to cisplatin and/or onalespib. Induction of apoptosis and DNA damage response were evaluated in both monotherapy and combination therapy groups.

**Results:**

Results demonstrate that onalespib enhances the efficiency of cisplatin in a dose-dependent manner. Tumor cells treated with both drugs displayed lower viability and a decreased migration rate compared to vehicle-control cells and cells treated with individual compounds. An increase of DNA double strand breaks was observed in both cisplatin and onalespib treated cells. The damage was highest and most persistent in the combination group, delaying the DNA repair machinery. Further, the cisplatin and onalespib co-treated cells had greater apoptotic activity compared to controls.

**Conclusion:**

The results of this study demonstrate that the reduced therapeutic efficacy of cisplatin due to drug-resistance could be overcome by combination treatment with onalespib. We speculate that the increased apoptotic signaling, DNA damage as well as the downregulation of HSP90 client proteins are important mechanisms promoting increased sensitivity to cisplatin treatment.

## Introduction

Cisplatin (cis-diamminedichloridoplatinum(II), CDDP) is one of the most commonly used antineoplastic drugs worldwide. The platinum-based compound has been in clinical use for more than 40 years and is a cost-effective first-in-line treatment against several solid cancers including ovarian, head and neck and testicular cancer (1). The main mechanism of action of cisplatin and other platinum-based analogs involves inter-or intra-strand crosslinks mediated by binding to reactive metal-binding sites on the DNA, primarily the N7 atoms of guanine and adenine in the major groove (2). These crosslinks disrupt DNA transcription and replication and can result in the induction of cytotoxic processes such as apoptosis. Furthermore, cisplatin is highly electrophilic and thus interacts with numerous nucleophilic non-DNA targets in the cytoplasm upon entering the cell. These interactions account for additional antineoplastic effects of the drug (2).

Though cisplatin is one of the most effective anticancer drugs, issues of acquired or innate resistance along with the serious adverse effects of the drug limit its curative potential (3, 4). However, the efficacy varies among the different types of cancer, in which ovarian and head and neck cancers present the greatest challenge. Development of cisplatin resistance is frequent, and linked to multiple mechanisms. One primary resistance mechanism is the reduction of uptake and accumulation of the compound in cancer cells (5). Although a large fraction of cisplatin is believed to enter the cell through passive diffusion, recent studies have indicated that copper transporters 1 and 2 (CTR1 and CTR2) are involved in the active transport of cisplatin (6). Studies have shown that cisplatin therapy downregulates CTR1 and CTR2, resulting in reduced uptake and subsequently decreased intracellular accumulation of cisplatin (7). Similarly, proteins involved in copper efflux, ATP7A and ATP7B, regulate the efflux of cisplatin, resulting in decreased intracellular cisplatin levels (8). Additionally, increased cisplatin-binding to glutathione S-transferase (GSH), metallothioneins and other cytoplasmic nucleophilic scavengers can decrease reactive intracellular levels of cisplatin (2, 5). Altered DNA-repair pathways further contribute to cisplatin resistance (4, 9). The primary repair mechanism utilized by cells following cisplatin-induced DNA damage is nucleotide excision repair (NER). NER involves more than thirty proteins but cisplatin resistance is most commonly associated with ERCC1, which is essential to catalyze the DNA excision step. High levels of ERCC1 have been associated with cisplatin resistant cancers, whereas low levels of ERCC1 are found in cisplatin sensitive cancers (10). Additionally, alterations in general stress response pathways including the heat shock response can promote cisplatin resistance (2, 5).

Heat shock proteins (HSP) are highly conserved molecular chaperones that play important roles in the formation and maturation of proteins involved in a wide diversity of cellular pathways, and subsequently have noticeable effects on the biology of normal and cancer cells. Among the HSP, HSP90 is a promising target in cancer therapy (11). HSP90 plays an essential role in signal transduction, conformational folding and cellular localization and stabilization of its client proteins (12, 13), which in turn are involved in processes such as transcriptional regulation, chromatin remodeling, cellular homeostasis, and DNA repair. So far, more than 300 HSP90 clients have been discovered. Among the clients are members of the epidermal growth factor receptor family (EGFR), signal transduction proteins (AKT and ERK) or DNA damage response proteins such as ATM (14). Many HSP90 client proteins are cancer-related, and elevated levels of HSP90 are often found in cancer. As a result, the malignancy is retained with the help of HSP90 and becomes particularly dependent on its activity, leading to an “HSP90 addiction” (15, 16). However, this dependency of HSP90 makes the cancer more susceptible to HSP90 inhibition. Therefore, inhibition of HSP90 offers the unique opportunity to overcome HSP90 dependency and to shut down several oncogenic processes simultaneously.

Several HSP90 inhibitors are currently undergoing clinical trials as cancer therapies, both as monotherapy and in combination with common antineoplastic therapies or radiation therapy (17, 18). HSP90 inhibitors mainly target the N-terminal ATPase on HSP90 and are able to displace ATP, blocking HSP90 function (11, 19). HSP90 inhibitors have been investigated as antineoplastic drugs since 1998, and in the intervening decades even more efficient inhibitors have been developed. Although promising on a preclinical level, the clinical usage of the first HSP90 inhibitors such as 17-AAG was limited due to issues with solubility, hepatotoxicity and the potential formation of toxic metabolites (20). Newer generations of HSP90 inhibitors such as AUY922, KW2478, STA-9090, and ONALESPIB387 display lower toxicities and improved function. Among them, onalespib (AT13387) is a potent second-generation compound, currently undergoing phase II studies in advanced solid tumors (13, 17). Studies have demonstrated potent radiosensitizing effects of onalespib both *in vitr*o and *in vivo*, an effect likely mediated by impairment of the DNA damage response (13, 21, 22). Here, combination therapy of onalespib and radiotherapy resulted in a substantial increase in DNA double breaks (DSBs) as well as delay in DNA repair measured by the DSB markers γH2AX and 53BP1 (22). These findings raise the question whether HSP90 inhibition may also enhance the cytotoxic effect of cisplatin, due to similarities between the effects of cisplatin and ionizing radiation on tumor cells.

The frequent development of cisplatin resistance in monotherapy has encouraged fruitful research on cisplatin combination therapies. Subsequently, cisplatin has become the backbone of several combination therapies for a wide range of solid tumors including bladder, cervical, ovarian, lung, gastric, breast, and head and neck cancers. However, combination therapy with HSP90 inhibitors is still under investigation and ongoing clinical trials are evaluating the combination of onalespib, cisplatin and radiotherapy (13, 21, 23). Combination treatments with cisplatin are of great interest, both due to its aforementioned wide range of activity, high initial level of activity and the ubiquity and low cost of treatment. Whereas there currently are many novel and highly advanced cancer drugs under investigation, many new compounds are exorbitantly expensive once they reach clinical use. This results in an unavailability for large sections of the worldwide patient population, resulting in an increased global and socio-economic gap in quality of cancer care. Focusing on restoring or enhancing the efficacy of widely available and affordable drugs by innovative use of combination therapy is therefore an attractive prospect for reducing this gap.

In the present study, we have evaluated whether therapy with the novel HSP90 inhibitor onalespib can potentiate the efficacy of cisplatin and reverse cisplatin resistance *in vitro*. We examined the efficacy of the drugs in H314, a head and neck squamous cell carcinoma cell line, and in the ovarian cancer cell lines SKOV3, A2780 and its cisplatin resistant clone A2780CIS. Furthermore, the underlying molecular mechanisms for the combination treatment were investigated.

## Materials and Methods

### Cell Lines and Culture Conditions

The human ovarian cancer cell line SKOV3 (doubling time 24 h) obtained from American Type Culture Collection (ATCC, Manassas, VA, United States) was cultured in RPMI 1640 medium (Biochrom GmbH, Berlin, Germany) supplemented with 10% fetal bovine serum (FBS) (Sigma Aldrich, St. Louis, United States), 2 mM L-glutamine (Biochrom GmbH) and antibiotics (100 IU penicillin and 100 μg/ml streptomycin, Biochrom GmbH) (24). The human head and neck squamous cell carcinoma cell line H314 (doubling time 34 h) was obtained from The European Collection of Authenticated Cell Cultures (ECACC, Salisbury, United Kingdom) and was cultured in Dulbecco’s Modified Eagle Medium/Ham’s F-12 medium (1:1, Biochrom GmbH) with the previously described supplements (25). The human ovarian cancer cell lines A2780 and the cisplatin resistant clone A2780CIS (doubling times of 18 h) were obtained from The European Collection of Authenticated Cell Cultures and cultured in RPMI 1640 medium with the previously described supplements (26). In order to retain cisplatin resistance for the clone, 1 μM cisplatin was added to the media every 2–3 passages. All four cell lines were incubated at 37°C with 5% CO_2_ and split two-to-three times a week using Trypsin/EDTA (Biochrom GmbH) when cells reached 80–90% confluency. All cell lines have been cultured for less than 3 months.

### Drug Preparation

Onalespib (Selleck Chemicals, Houston, TX, United States) was dissolved in DMSO to a stock concentration of 61.0471 mM and stored in aliquots at −20°C. The stock concentration of cisplatin from EBEWE Pharma (Unterach am Attersee, Austria) was 1 mg/ml and was stored at room temperature. Both cisplatin and onalespib were diluted further in complete media for assay dependent concentrations. The final DMSO concentration was 0.005% (v/v) for 3000 nM Onalespib, 0.0002% (v/v) and 0.00008% (v/v) for 100 nM for 50 nM, respectively.

### XTT Cell Viability Assays

A defined number of cells were seeded in flat-bottomed 96-well plates (SKOV3: 2000 cells/well, H314: 15000 cells/well, A2780: 2000 cells/well and A2780CIS 3000 cells/well) and incubated at 37°C and 5% CO_2_ for 48 h prior to drug incubation with 0–3000 nM onalespib and 500 nM, 10 μM and 25 μM cisplatin. Cells were incubated at 37°C and 5% CO_2_ for 24 h or 72 h. XTT Activation Reagent and XTT Reagent were added according manufacturer’s instructions (American Type Culture Collection protocol 30–1011 K, Manassas, VA, United States). Plates were incubated for 4 h (SKOV3, A2780, and A2780CIS) and 3 h (H314) at 37°C and 5% CO_2_ and absorbance was measured using a BioMark Microplate Reader (Bio-Rad Laboratories AB, Solna, Sweden). Significance was determined using two-way ANOVA followed by Sidak’s multiple comparison’s test. The number of replicates within each experimental group was 3 or more. Each experiment was repeated three times.

### Clonogenic Survival Assay

Clonogenic survival assays were performed as described previously (27). In short, SKOV3, H314, A2780, and A2780CIS cells were seeded in 6-well plates and treated 24 h after seeding, with either cisplatin (100 and 250 nM) or onalespib (50 and 100 nM) as well as with combined treatment (100 nM of cisplatin with 50 nM of onalespib, 100 nM of cisplatin with 100 nM of onalespib, 250 nM of cisplatin with 50 nM onalespib, and 250 nM of cisplatin with 100 nM onalespib). After a drug incubation time of 24 h, the medium was replaced with complete media corresponding to the cell line and cells were incubated until colonies of >50 cells/colony were formed. After colony formation time (H314: 20 days, SKOV3: 10 days, A2780: 14 days, A2780CIS: 14 days), cells were fixed with 95% ethanol and stained with crystal violet. Colonies containing >50 cells were scored manually and plating efficiency (PE) and survival fraction (SF) were calculated. One-way ANOVA followed by Tukey’s multiple comparison’s test determined significance. Data were expressed as mean ± SD and *p* < 0.05 considered to be statistically significant. The number of replicates within each experimental group was 3. Each experiment was repeated three times.

### Wound Healing Assay

Wound healing assay was performed as per published protocol (28). Briefly, cells were seeded in 48 well-plates (H314) or 6 well-plates (SKOV3). After 24 h, the confluent cell monolayer was scratched with a p10 pipette tip and was immediately treated with either cisplatin (100, 250, and 500 nM), onalespib (50 and 100 nM) or combinations thereof. Images from the same scratch location (three areas for each concentration) were obtained directly after scratching, 8 h and 24 h for SKOV3 cells and 24, 48, and 72 h of incubation for H314 cells using an inverted microscope Nikon Diaphot (Nikon, Japan) mounted with a Canon EOS 700D camera (Canon Inc., Japan). Migration distance was measured and analyzed using ImageJ 1.51k software (NIH, Bethesda, MD, United States). One-way ANOVA followed by Tukey’s multiple comparison’s test determined significance. Data were expressed as mean ± SD and *p* < 0.05 considered to be statistically significant. The number of replicates within each experimental group was three. Each experiment was repeated three times.

### *Trans-*Well Migration Assay

*Trans-*well migration assay was performed using 24-well plates with inserts of 8 μm filter (Thermo Fisher Scientific, Sweden). Cells were starved for 24 h before adding cell suspension in FBS^–^ media (1 × 10^5^ cells/chamber) and 250 nM cisplatin and/or 50 and 100 nM onalespib into the upper chamber with a total volume of 100 μl. 500 μl 10% FBS containing media was placed in lower chamber. After overnight incubation at 37°C, remaining cells in the upper chamber were removed and the migrated cells on the bottom side of the filter were fixed in 99.7% ethanol for 10 min and stained with 1% crystal violet for 2 min. Five images of each insert were captured with microscope at ×200 magnification. and ImageJ (version 2.0, NIH, United States) was used for manual scoring of the migrated cells and for analysis. One-way ANOVA followed by Tukey’s multiple comparison’s test determined significance. The experiments were repeated at least two times (*N* > 2).

### Western Blotting

After a 24 h or 96 h drug incubation with either 250 or 500 nM cisplatin, 50 or 100 nM onalespib or combinations thereof, whole cell lysates of SKOV3 and H314 cells were prepared as follows: cells were washed once with 1x cold PBS and incubated with Pierce^®^ IP Lysis Buffer containing 1x phosphatase and protease inhibitor cocktail (Thermo Fisher Scientific, Sweden) for 15 min on a tilting ice bed. The cell lysates were centrifuged for 15 min at 15000 rpm at 4°C and subsequently stored at −20°C. Following protein quantification (Pierce BCA Protein Assay Kit, Thermo Scientific, Sweden) samples were separated on an SDS-PAGE using 4–12% Bis-Tris gels in MES or MOPS SDS running buffer or 3–8% Tris–Acetate gels in Tris–Acetate SDS running buffer (Novex^TM^, NuPAGE^®^, Invitrogen, Thermo Fisher Scientific, Sweden). Thereafter, the separated proteins were transferred to a PVDF membrane (Merck Millipore, Darmstadt, Germany) using wet transfer for 2 h with the constant voltage of 100 V at room temperature using an insert ice block for cooling. The membranes were blocked in Western Blot fluorescent Blocking Buffer (ThermoFisher Scientific, Sweden) or 5% bovine serum albumin in PBS-Tween (0.1%) for 60 min. The membranes were incubated with the primary antibody targeting EGFR (2232S Rabbit polyclonal antibody, Cell Signaling Technology, United States), AKT1,2,3 (ab179463 Rabbit monoclonal antibody, Abcam, United Kingdom), Anti-AKT (phospho T308) (Rabbit polyclonal antibody, Abcam, United Kingdom), Anti-AKT1 + AKT2 + AKT3 (phospho Y312 + Y315 + Y316) (Rabbit polyclonal antibody, Abcam, United Kingdom), H2AX (Rabbit polyclonal antibody, Abcam, United Kingdom), γH2AX (Rabbit monoclonal antibody, Abcam, United Kingdom and Mouse monoclonal antibody, JBW clone 301, Millipore GmbH, Germany), ATM (Rabbit monoclonal antibody, Abcam, United Kingdom) and DNA-PKcs (Rabbit monoclonal antibody, Abcam, United Kingdom), overnight at 4°C. Beta-actin (Mouse monoclonal, Sigma Aldrich, Sweden) or sodium-potassium ATPase (ab76020, Abcam, United Kingdom) was used as loading control. The following day, the membranes were incubated with secondary antibodies (Invitrogen) in 0.1% PBS-Tween for 60 min and developed using the Amersham^TM^ Imagequant^TM^ 800 (ThermoFisher Scientific, Sweden). The bands were quantified by using ImageJ software. One-way ANOVA followed by Tukey’s multiple comparison’s test determined significance where *p* < 0.05 was considered significant. The experiments were repeated at least three times (*N* = 3).

### Analysis of Apoptosis via Flow Cytometry

SKOV3 and A2780CIS cells were plated in T25 flasks 24 h before drug exposure. Afterward, samples were treated with 37°C warm media mixed with 100 nM onalespib, 500 nM cisplatin, or a combination of onalespib and cisplatin for 96 h before flow cytometry. Harvested cells were washed in cold PBS and stained with propidium iodide (PI) and Alexa Fluor 488 annexin V (Alexa Fluor^®^488 Annexin V/Dead Cell Apoptosis Kit with Alexa Fluor 488 annexin V and PI for flow cytometry, ThermoFisher Scientific, Sweden) according to manufactures instructions. CellEvent^TM^ Caspase-3/7 Green Flow Cytometry Assay Kit (Thermo Fisher Scientific, Sweden) was used to analyze caspase 3/7 activity. Caspase activity inhibition on SKOV3 and A2780CIS apoptosis were evaluated by pan-caspase inhibitor z-VAD-FMK (Selleckchem, Germany). Cells were pretreated with or without 20 μM z-VAD-FMK for 1 h followed by incubation with 500 nM cisplatin and 100 nM onalespib. Apoptotic cells were visualized using a CytoFLEX (Beckman Coulter, Krefeld, Germany). Obtained data were analyzed by FlowJo^TM^ Software for Windows (Version 10.6.1. Becton, Dickinson and Company, Ashland, OR, United States). One-way ANOVA followed by Tukey’s multiple comparison’s test determined significance, where *p* < 0.05 was considered significant. The number of replicates within each experimental group was two. Each experiment was repeated three times.

### Cell-Cycle Distribution Analysis via Flow Cytometry

After 96 h exposure to 500 nM cisplatin, 100 nM onalespib, or the combination thereof SKOV3 and A2780CIS cells were harvested and washed with ice-cold PBS followed by resuspension in 0.5 mL PBS. Cells were fixed by adding 5 mL of ice-cold 70% EtOH drop-wise and incubated at −20°C overnight. Afterwards, the cells were centrifuged at 1200 rpm for 5 min and washed once with 2 mL ice-cold PBS. After removing the supernatant, cells were centrifuged again at 1200 rpm for 5 min followed by removing the supernatant and adding 0.5 mL RNase (100 μg/mL) and 100 μL of PI (50 μg/mL). The cells were incubated for 30 min at RT in the dark before analysis using a CytoFLEX (Beckman Coulter, Krefeld, Germany). The data analysis and peaks recognition performed in FlowJo^TM^ Software for Windows (Version 10.6.1. Becton, Dickinson and Company, Oregon, United States).

### Analysis of γH2AX and 53BP1 Expression via Immunofluorescence Staining (Confocal Microscopy)

SKOV3 and A2780CIS cells were seeded in 4-well cell culture chamber slides (Nunc A/S, Roskilde, Denmark) and incubated for 24 h before drug treatment for a confluency of 60% prior to start of treatment. Thereafter, cells were incubated with either mono-or combination treatments of 500 nM cisplatin and 100 nM onalespib for 96 h. After treatment, slides were washed with 1x PBS followed by 99% methanol fixation at −20°C. Cell membrane permeability was induced by ice-cold acetone exposure for 10–15 s (Millipore, Merck, United States). Non-specific protein blocking was performed in 10% FBS-PBS for 60 min at room temperature to reduce background interference. Cells were incubated with primary Rabbit anti-53BP1 (Abcam, Cambridge, United Kingdom) and mouse anti-γH2AX (EMD Millipore Merck Darmstadt, Germany) antibodies overnight at 4°C and secondary antibody incubation [master mix of Alexa flour 488 (ab150117, Abcam, Cambridge, United Kingdom) and Alexa flour 555 (ab150086, Abcam, Cambridge, United Kingdom)] were done the following day for 60 min in the dark. DAPI (ThermoFisher Scientific, Sweden) was used for nucleus staining in the dark for 2 min followed by 10 washes with 1x PBS and milli-Q water. After air-drying, the VectaShield (Vectorlabs, Burlingame, CA, United States) were mounted on slides and covered with a coverslip. Slides were imaged at three randomly chosen fields of view with a Zeiss LSM 700 confocal microscope (Zeiss, Oberkochen, Germany). The accuracy of foci image acquisition was confirmed by Z-stacking with different magnifications. Image processing and foci counting were performed using Image J software. One-way ANOVA followed by Tukey’s multiple comparison’s test determined significance, where *p* < 0.05 was considered significant. The experiments were repeated three times (*N* = 3).

## Results

### Cisplatin and Onalespib Monotherapy Decreases Viability of Cancer Cells While Co-treatment Potentiates the Effects

**FIGURE 1:**
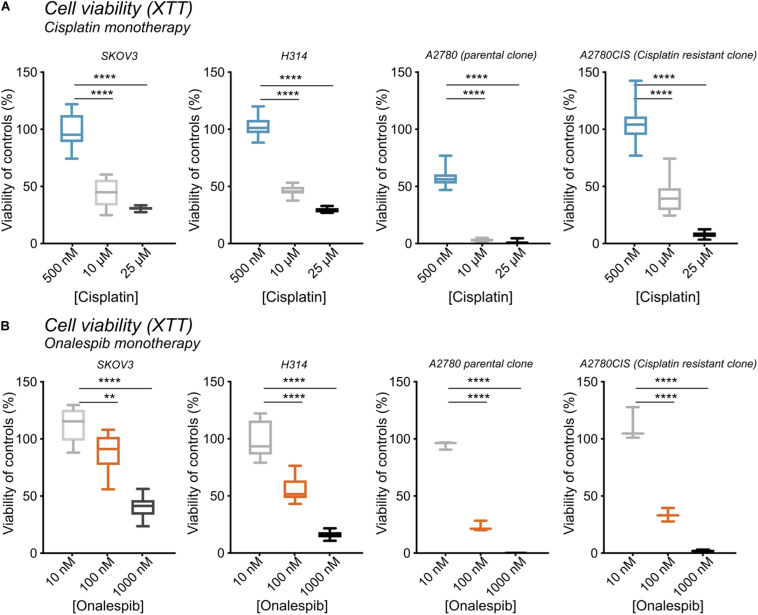
**(A)** XTT cell viability from left to right of SKOV3, H314, A2780, and A2780CIS cells following treatment with 500 nM, 10 μM, or 25 μM cisplatin normalized to untreated controls. **(B)** XTT cell viability from left to right of SKOV3, H314, A2780, and A2780CIS cells following treatment with 10, 100, or 1000 nM onalespib normalized to untreated controls. *N* = 3, error bars represent SD. **p < 0.01, ****p < 0.0001.

The growth inhibitory effects of monotherapy with cisplatin and the HSP90 inhibitor onalespib were first assessed in ovarian cancer cell lines SKOV3, A2780, and A2780CIS cells as well as head and neck cancer cells (H314) following both 24 h and 72 h drug incubations (Figures 1A,B and Supplementary Figure 1). Increasing concentrations of cisplatin decreased the viability as measured by XTT metabolic assay in all cell lines in a concentration dependent manner (Figure 1A). In the XTT assays 500 nM cisplatin did not affect the viability of SKOV3, H314, or A2780CIS cells using a drug incubation time of 72 h. However, the A2780 cells demonstrated a decrease in viability by about >50% as a result of a 72 h drug incubation time. Concentrations of 10 μM decreased the viability by 57.5% and 53% and 25 μM cisplatin by 70 and 71% in SKOV3 and H314 cells, respectively. Similarly, the A2780CIS cells was greatly affected by 10 and 25 μM cisplatin, resulting in a decrease in viability by 58 and 98%, respectively.

Increasing concentrations of onalespib also decreased the viability as measured by XTT. 100 nM onalespib decreased the viability by 11% and 45%, and 1 μM onalespib by 60% and 90% in SKOV3 and H314 cells in the XTT analysis, respectively (Figure 1B). The A2780 and A2780CIS cells were more sensitive to onalespib monotherapy than SKOV3 and H314 cells. Following incubation with 100 nM of onalespib, the viability of A2780 and A2780CIS cells decreased by 80% and 70%, respectively. 1 μM of onalespib resulted in a nearly undetectable signal, nearing 100% decrease in viability.

**FIGURE 2:**
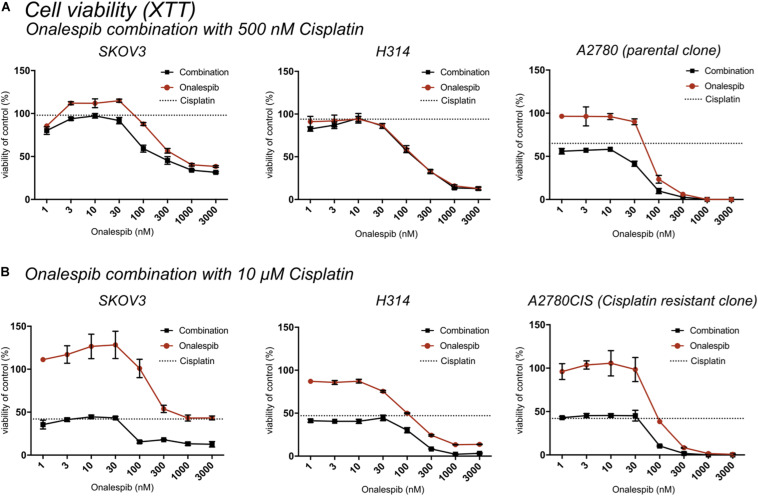
**(A)** XTT cell viability normalized to untreated controls of SKOV3, H314, and A2780 cells treated with 0–3000 nM onalespib or the combination of onalespib with 500 nM cisplatin **(A)** 72 h post treatment. **(B)** XTT cell viability normalized to untreated controls of SKOV3, H314 and A2780CIS cells treated with 0–3000 nM onalespib or the combination of onalespib with 10 μM cisplatin at 72 h post treatment. *N* = 3, error bars represent SEM. Dotted lines represent the viability of cisplatin monotherapy at either 500 nM or 10 μM.

At 24 h, the viability of SKOV3 and H314 cells was not significantly affected by cisplatin monotherapy at either concentration (Supplementary Figure 1A, dotted lines), whereas onalespib monotherapy resulted in decreased viability at concentrations exceeding 100 nM for both cell lines (Supplementary Figure 1A). Analysis of the later time point (72 h) demonstrated greater effects in both SKOV3, H314 and A2780CIS cells treated with 10 μM cisplatin, where viability had decreased to less than 50% of untreated controls, compared with 90–100% of untreated controls at 24 h post treatment (dotted lines in Figures 2A,B). Similarly, the effects of onalespib increased over time, resulting in significantly decreased viability of samples treated with 30 nM or higher (Supplementary Figures 1A, 2A,B).

The potency of the combination of cisplatin and onalespib was greater at the later time point (72 h) and the high cisplatin concentration (10 μM). In these samples, a significant decrease in viability was measured in all combination treated samples compared to monotherapy in both cell lines. In H314 cells, the combination of 10 μM of cisplatin and doses ≥300 nM onalespib resulted in nearly indistinguishable absorbance levels (13% for onalespib monotherapy at 1000 and 3000 nM and 2% for combination therapy at the same concentrations), effectively reducing survival of the cells to near zero. For SKOV3 and A2780CIS cells, the same pattern was observed, resulting in a viability of the combination of 10 μM cisplatin and 100 or 300 nM onalespib below 15% (Figure 2B). Interestingly, H314 samples treated with 500 nM of cisplatin were unaffected and there were no differences between onalespib monotherapy and combination treated samples. SKOV3 and A2780 cells treated with 500 nM cisplatin were affected by the combination therapy, with significant differences between onalespib monotherapy and combination treated samples at onalespib concentrations ≤100 nM (Figure 2A).

### Combination Therapy Significantly Impairs Clonogenic Survival

**FIGURE 3:**
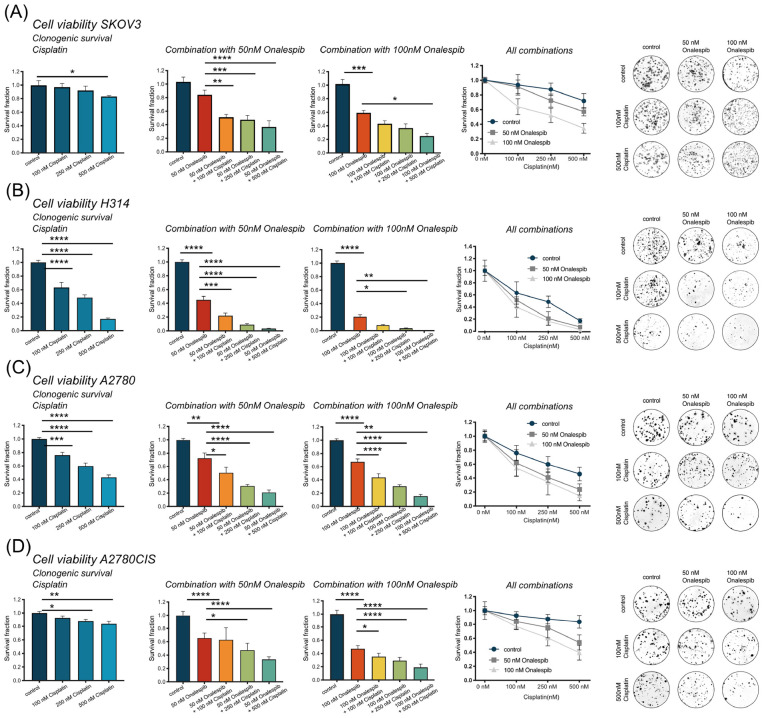
**(A)** Clonogenic survival of SKOV3 cells treated with 0, 100, 250, and 500 nM cisplatin as monotherapy or in combination with 50 or 100 nM onalespib. Note that combination samples are normalized to 0, 50, or 100 nM of onalespib to compensate for the effect on survival by onalespib alone. **(B)** Clonogenic survival of H314 cells treated with 0, 100, 250, and 500 nM cisplatin as monotherapy or in combination with 50 or 100 nM onalespib. Note that combination samples are normalized to 0, 50, or 100 nM of onalespib to compensate for the effect on survival by onalespib alone. **(C)** Clonogenic survival of A2780 cells treated with 0, 100, 250, and 500 nM cisplatin as monotherapy or in combination with 50 or 100 nM onalespib. **(D)** Clonogenic survival of A2780CIS cells treated with 0, 100, 250, and 500 nM cisplatin as monotherapy or in combination with 50 or 100 nM onalespib. Note that combination samples are normalized to 0, 50, or 100 nM of onalespib to compensate for the effect on survival by onalespib alone. *N* = 3, error bars represent SD. *p < 0.05, **p < 0.01, ***p < 0.001, ****p < 0.0001.

The efficacy of cisplatin and onalespib combination therapy was also studied in clonogenic survival assays. The highest concentration of cisplatin (500 nM) significantly decreased the survival fraction of SKOV3, H314, A2780, and A2780CIS cells (Figures 3A–D, left hand graph). Monotreatment with 100 nM onalespib decreased the survival of H314, A2780, and A2780CIS cells, but not of SKOV3 cells. However, the combination of cisplatin and onalespib significantly affected the survival fractions of all cell lines compared to untreated controls and cells treated with onalespib alone (Figures 3A–D middle and right-hand graphs). Generally, A2780 and H314 cells were more sensitive to the treatments than SKOV3 and A2780CIS cells (Figures 3A–D, middle). Cisplatin in combination with 50 nM onalespib displayed a clear increase in effect, where all survival fractions of all combination treatments were significantly decreased compared to both untreated controls and onalespib monotherapy. For 100 nM onalespib, the difference between combination treatment and monotreatment was lower, due to the high effect of onalespib alone. Here, all four cell lines treated with 100 nM onalespib were significantly affected compared to untreated controls (*p* ≤ 0.001). Furthermore, the combination of 500 nM cisplatin and 100 nM onalespib was significantly decreased from onalespib monotherapy in all cell lines (*p* ≤ 0.05). Combination therapy of H314 and A2780 cells decreased the survival fraction in all tested combinations (*p* ≤ 0.0001). Besides the effects on the survival fraction, the shape and size of the colonies was affected by the drug treatment. In general, cells treated with increasing drug combinations showed smaller colony sizes. Furthermore, colonies of the combination treatment groups were more irregular in shape (Figures 3A–D, right hand).

### Combination Therapy of Cisplatin and Onalespib Delayed Wound Healing and Decreased the Migration of Cancer Cells

#### Wound Healing Assay

**FIGURE 4:**
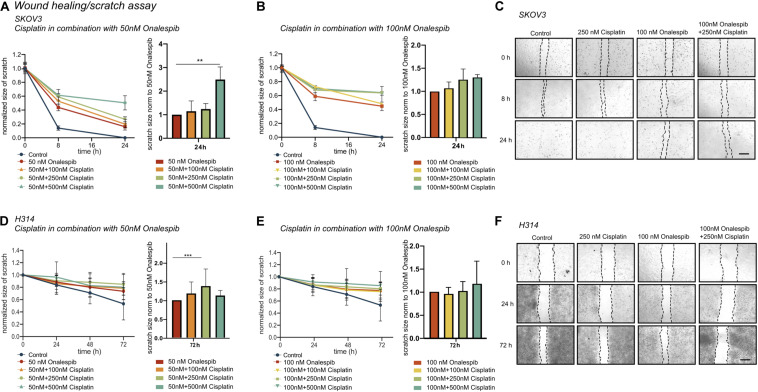
**(A)** Effect of 50 nM onalespib and the combination with 100, 250, or 500 nM cisplatin and **(B)** effect of 100 nM onalespib and the combination with 100, 250, or 500 nM cisplatin at 24 h on the wound healing ability of SKOV3 cells. Note that in figure [**(A)** – right] and [**(B)** – right], wound/scratch sizes are normalized to 50 nM onalespib **(A)** or 100 nM onalespib **(B)**. **(C)** Representative images of SKOV 3 wound/scratch of control, 250 nM cisplatin, 100 nM onalespib and the combination at 0, 8, and 24 h post scratch. **(D)** effect of 50 nM onalespib and the combination with 100, 250, or 500 nM cisplatin and **(E)** effect of 100 nM onalespib and the combination with 100, 250, or 500 nM cisplatin at 24 h on the wound healing ability of H314 cells at 72 h. Note that in figure [**(D)** – right] and [**(E)** – right], wound/scratch sizes are normalized to 50 nM onalespib **(D)** or 100 nM onalespib **(E)**. **(F)** Representative images of H314 wound/scratch of control, 250 nM cisplatin, 100 nM onalespib and the combination at 0, 24, and 72 h post scratch. *N* = 3, error bars represent SD. **p < 0.01, ***p < 0.001. The size bar in **(C,F)** corresponds to 4 μm.

To further study whether onalespib treatment can augment cisplatin therapy, the migration capacity of SKOV3 and H314 cells was studied in wound healing assays. Cisplatin monotherapy did not affect the wound healing ability of SKOV3 cells (Supplementary Figure 1C), whereas a dose-dependent decrease in migration capacity/healing was observed for onalespib (50 and 100 nM) monotherapy samples (Figures 4A–C). The combination of 50 nM onalespib with 500 nM cisplatin (Figure 4A) resulted in a significant (*p* ≤ 0.01) delay in wound healing in compared to either monotherapy at 24 h post start of the assay (Figure 4B, bar chart). A similar trend in inhibitory effect was observed for the combination with 100 nM onalespib, though not statistically significant (Figures 4B,C). H314 cells did not migrate as fast as SKOV3 cells. Therefore, this cell line was followed up to 72 h. H314 cells were unaffected by cisplatin monotherapy (Supplementary Figure 1C) after 72 h, but onalespib monotherapy displayed a dose-dependent decrease in healing. However, the onalespib combination therapy had a more potent effect compared to monotherapy in the H314 cells (Figures 4D–F). The combination of cisplatin and onalespib resulted in a significant delay in wound healing for the combination of 50 nM of onalespib with 250 nM cisplatin (*p* ≤ 0.001), but paradoxically not for 500 nM cisplatin. Similarly to SKOV3 cells, the greatest inhibitory effect was seen in the 100 nM onalespib and 500 nM cisplatin group, though the difference to monotherapy was not statistically significant (Figure 4E). Representative images of the scratches are shown in Figure 4C for SKOV3 cells and in Figure 4F for H314 cells.

#### *Trans-*Well Migration Assay

Since the wound healing assays measure a mixture of migration and proliferation, the migrating potential of serum-starved SKOV3 and H314 cells was specifically investigated using *trans-*well migration assays with a pore size 0.8 μm. Increasing doses of cisplatin decreased the number of migrated SKOV3 cells, with significant decrease measured at 250 nM (63.3 ± 9.2%) and 500 nM cisplatin (17.1 ± 2.1%) compared to control cells. Similarly, increasing doses of onalespib resulted in a significantly lower number of migrated SKOV3 cells at concentrations ≥100 nM (54.1 ± 8% and 21 ± 3.7% for 100 and 200 nM, respectively) compared to untreated controls. Moreover, the combination of cisplatin and onalespib resulted in additionally lowered migration (Table 1), although none of the tested combination treatments resulted in significant changes compared to monotherapies. Microscopic images of the migrated cells are displayed in Supplementary Figure 1D. H314 cells were unable to migrate in the *trans-*well migration assays, where as few as ten cells had migrated after 48 h in the control samples (data not shown).

**TABLE 1:** Mean, SEM, and 95% confidence intervals (CI) of migrated SKOV3 cells treated with 250 or 500 nM cisplatin, 50 or 100 nM onalespib or the combination during the *trans-*well migration assays, *N* = 3.

Treatments	Mean of migrated cells ± SEM	95% Cl
Control	100 ± 4	8.4
250 nM Cisplatin	63.3 ± 9.2	19.2
500 nM Cisplatin	17.1 ± 2.1	4.8
50 nM Onalespib	80.6 ± 5.5	11.9
100 nM Onalespib	54.1 ± 8	18.1
200 nM Onalespib	21 ± 3.7	8.5
250 nM Cisplatin + 50 nM Onalespib	65.3 ± 9	19.4
250 nM Cisplatin + 100 nM Onalespib	51.4 ± 7	16
250 nM Cisplatin + 200 nM Onalespib	23 ± 4	9.7
500 nM Cisplatin + 50 nM Onalespib	24.2 ± 4	13.8
500 nM Cisplatin + 100 nM Onalespib	26.5 ± 7	20.3
500 nM Cisplatin + 200 nM Onalespib	18.2 ± 2	4.6

### Onalespib and Cisplatin Treatment Downregulate Cell Signaling and HSP90 Client Proteins

**FIGURE 5:**
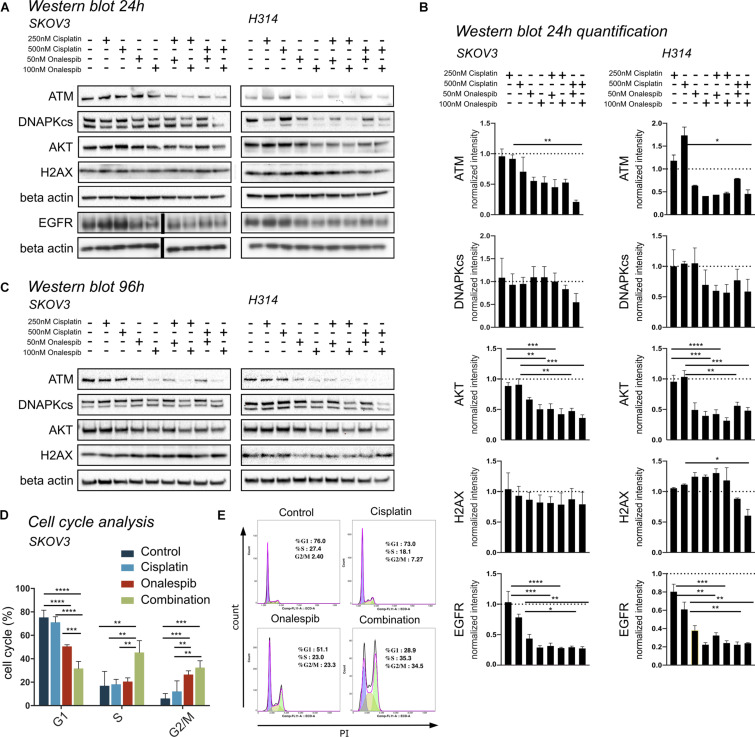
**(A)** Representative Western blot membrane of the analysis of ATM, DNAPKcs, AKT, H2AX, and EGFR expression levels of SKOV3 and H314 cells following 24 h incubation with cisplatin and onalespib and their combinations. Note for EGFR analysis is from a separate membrane and the beta actin below is the loading control for this particular membrane. The black bar inserted in the middle of the membrane is due to removal of an overexposed size marker **(B)** Quantification of Western blot at 24 h incubation of SKOV3 and H314 cells. **(C)** Representative Western blot membrane of the analysis of ATM, DNAPKcs, AKT and H2AX expression levels of SKOV3 and H314 cells following 96 h incubation with cisplatin and onalespib and their combinations. **(D,E)** Cell cycle distribution in SKOV3 cells after exposure to cisplatin, onalespib, and their combinations as percentage of cells in G1, S, and G2M phases. **(E)** Representative flow cytometry graphs. Combination treatment led to cell cycle arrest and elevated G2/M peak compare to monotherapy groups. *N* = 3, error bars represent SEM. *p < 0.05, **p < 0.01, ***p < 0.001, ****p < 0.0001.

Western blotting was used to study the effect of onalespib and cisplatin on HSP90 client proteins, downstream signaling cascades and DNA damage response proteins in SKOV3 and H314 cells (Figures 5A–C). In order to investigate DNA damage response, the expression of the repair proteins ATM and DNAPKcs were investigated. Treatment with onalespib reduced ATM and DNAPKcs expression, most pronounced at highest concentrations (100 nM onalespib) with and without cisplatin treatment (Figures 5A,B). Incubation with onalespib reduced ATM expression to a high degree, and no band was observed in H314 cells at highest concentrations (Figure 5C). To investigate whether the non-phosphorylated form of the DNA double strand break marker γH2AX is changed by onalespib and / or cisplatin, we examined the expression of the histon H2AX. At the 24 h time point, the level of H2AX expression was nearly constant for both cell lines in all treatment groups and in the control. At 96 h, a slight increase in the H2AX level was found for both cell lines, but not significant (Figure 5C).

The AKT expression levels for both cell lines decreased significantly in the onalespib and the combination treatment group compared to control and cisplatin monotherapy. For SKOV3 cells, only the combination of 250 nM cisplatin and 100 nM onalespib resulted in a significant decrease compared to cisplatin monotherapy (Figures 5A,B). For H314 cells, all combination treatments showed a significantly lower AKT expression compared to cisplatin monotherapy. For both cell lines, pAKT was not detected by Western blot at any time point (data not shown).

Cisplatin monotherapy at concentration <500 nM did not affect the expression of EGFR in both SKOV3 and H314 cells, whereas monotherapy with 50 nM and 100 nM onalespib significantly downregulated EGFR levels in a concentration-dependent manner (Figures 5A,B).

### Combination Therapy With Cisplatin and Onalespib Leads to G2/M Phase Arrest

Flow cytometric analysis of cell cycle distribution of SKOV3 cells using PI staining after exposure to 500 nM cisplatin, 100 nM onalespib, or combination for 96 h showed increasing number of cells in G2/M phase (32.5%) in the combination group compared to the monotreatment groups (onalespib 26.6% and cisplatin 12.1%) (Figures 5D,E). The percentage of cells in the G2/M phase of the combination group was significantly (*p* ≤ 0.01) higher than the cisplatin and (*p* ≤ 0.001) control group (6%). The percentage of cells in the S phase was significantly (*p* ≤ 0.01) higher in the combination group (45%) compared to onalespib (20.5%) and cisplatin (18.2%) and control (20%) samples. The G1 phase was decreased from 73.9% in the control group to 71.1% in the cisplatin group followed by 50.5% in the onalespib group and 31.7% in the combination group. Representative histograms are presented in Figure 5E.

### Combination Therapy of Cisplatin and Onalespib Increased Apoptotic Activity in Cancer Cells

**FIGURE 6:**
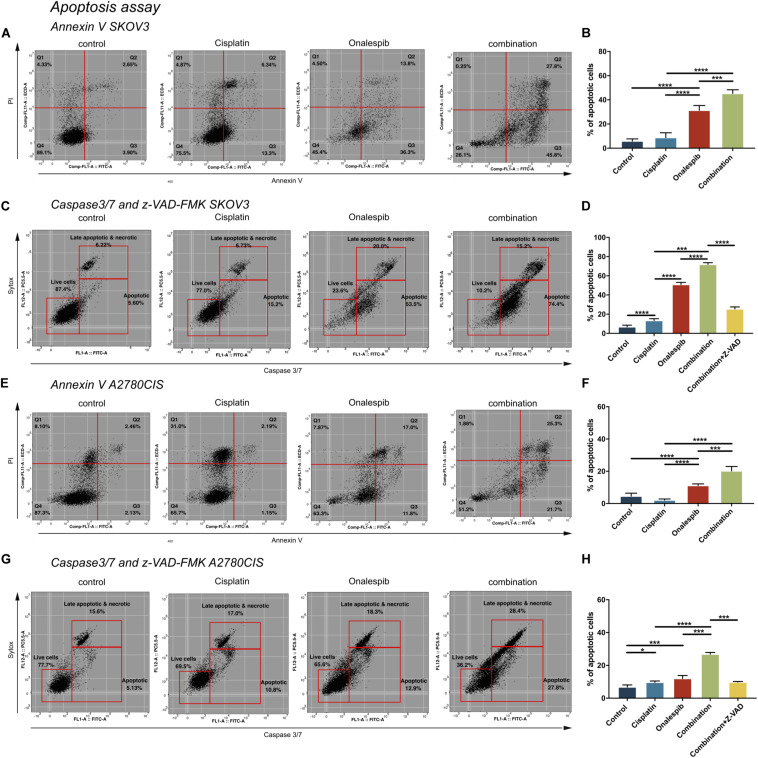
Flow cytometric analysis of apoptosis of untreated controls, 500 nM cisplatin, 100 nM onalespib and the combination using Annexin V/PI staining of **(A,B)** SKOV3 and **(E,F)** A2780CIS cells following 96 h incubation. The lower left square shows the percentage of live cells, the lower right square shows the percentage of apoptotic cells and the upper right square shows late apoptotic and necrotic cells. Flow cytometric analysis of caspase 3/7 of untreated controls, 500 nM cisplatin, 100 nM onalespib and the combination of **(C,D)** SKOV3 and **(G,H)** A2780CIS cells after 96 h incubation. Note that in **(C,D,G,H)**, the effects of combination therapy were also evaluated in combination with 20 μM of the z-VAD-FMK pan-caspase inhibitor. The lower left square shows the percentage of live cells, the lower right square shows the percentage of apoptotic cells and the upper right square shows late apoptotic and necrotic cells. *N* = 3, error bars represent SEM. *p < 0.05, ***p < 0.001, ****p < 0.0001.

SKOV3 and A2780CIS cells were treated with 500 nM cisplatin and 100 nM onalespib monotherapy and combination therapy for 96 h to investigate cell apoptosis by flow cytometry (Figure 6). Mean fluorescence intensity graphs are presented in Supplementary Figure 2.

Annexin V, a specific apoptotic cell membrane marker, revealed increased levels of apoptotic cells for both cell lines following all treatments (Figures 6A,B,E,F). In SKOV3 cells, combination treatment significantly elevated Annexin V activity to 45% compared to 31% in the onalespib treated group (*p* = 0.0003) and 9% in the cisplatin group (*p* < 0.0001). Similarly, albeit somewhat lower for A2780CIS cells than SKOV3 cells, Annexin V activity was significantly elevated to 20% in the combination group compared to 11% in the onalespib and 2% in the cisplatin monotherapy groups (*p* < 0.0001). The apoptotic response in the onalespib group was significantly higher than cisplatin treated and control cells (*p* < 0.0001) (Figures 6E,F).

To further characterize the apoptotic activity flow cytometric analysis of caspase 3/7-sytox and the pan-caspase inhibitor z-VAD-FMK were performed (Figures 6C,D,G,H). Similarly to the Annexin V results, apoptotic activation was greatest in SKOV3 cells compared to A2780CIS cells. In SKOV3 cells, caspase 3/7 increased significantly to 72% in the combination group compared to 51% and 13% in the onalespib and cisplatin monotherapy groups, respectively (*p* < 0.0001). Caspase activity in onalespib monotherapy samples was significantly higher than in cisplatin monotherapy samples (*p* < 0.0001). Treatment with the z-VAD-FMK pan-caspase inhibitor significantly reduced apoptotic activity to 25% in the combination group (*p* < 0.0001) (Figure 6C and Supplementary Figure 2E). The percentage of caspase 3/7 positive A2780CIS cells was significantly increased to 27% in the combination group compared to 12% in onalespib and 10% in cisplatin monotherapy groups (*p* < 0.0001). Here, the pan-caspase inhibitor significantly inhibited apoptotic activity to 10% in the combination group (*p* < 0.0001) (Figure 6H and Supplementary Figure 2J).

### Onalespib and Cisplatin Combination Treatment Increased Number of DNA Double Strand Breaks

**FIGURE 7:**
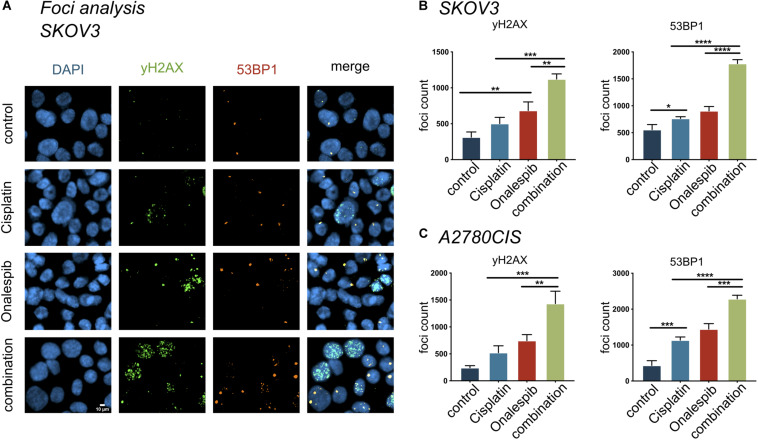
The compartmentalization of γH2AX, 53BP1 foci in SKOV3 cells *in vitro* exposed to mono- and combined treatment of cisplatin and onalespib. **(A)** Representative high-resolution images of γH2AX and 53BP1 foci units formation in SKOV3 cells *in vitro* exposed to 500 nM cisplatin, 100 nM onalespib, or a combination of cisplatin and onalespib for 96 h. The images panel demonstrate green stain for γH2AX, orange for 53BP1, and blue for stained nuclei with DAPI **(B,C)**. *N* = 3, foci counts are presented as the mean @ SD, *p < 0.05, **p < 0.01, ***p < 0.001, ****p < 0.0001.

The effect of onalespib and cisplatin combination treatment on the induction of DNA damage was studied by confocal microscopy through γH2AX foci and 53BP1 foci analyses of 300 SKOV3 and A2780CIS cells per replica (Figure 7). As demonstrated in Figure 7A, exposure of SKOV3 and A2780CIS cells to 500 nM cisplatin induced DSBs as measured by the number of γH2AX and 53BP1 foci. Monotherapy with 100 nM onalespib significantly increased the DNA damage, as measured by number of foci. In SKOV3 cells, the total number of 53BP1 foci was higher than that of γH2AX in all samples (Figures 7A,B). The number of 53PB1 foci was significantly elevated in the combination compared to onalespib and cisplatin monotherapy (*p* < 0.0001). The number of γH2AX foci significantly increased in the combination compared to onalespib (*p* = 0.0015) and cisplatin (*p* = 0.0001) monotherapy. The same DSBs induction trend was seen in A2780CIS cells. The combination produced significantly greater numbers of 53PB1 foci than onalespib (*p* = 0.0001) and cisplatin (*p* < 0.0001) monotherapy. The γH2AX foci number significantly increased in the combination therapy compared to onalespib (*p* = 0.0017) and cisplatin (*p* = 0.0002) monotherapy (Figure 7C).

Foci analysis was also performed on H314 cells, however, due to its different growth phenotype (in clusters, spheroid like) it was not possible to get quantifiable data on foci numbers (data not shown).

## Discussion

Forty years after the introduction of cisplatin, it remains a cornerstone cancer drug, widely used as a first-in-line treatment in many solid cancers. The initial response to cisplatin is high, as for all platinum-based drugs. The majority of patients will relapse with cisplatin-resistant disease, however, as rapid resistance development is one of the main limitations of cisplatin and related platinum-based analogs. Additional clinically important limitations include severe adverse side effects, which restrict the possibility of achieving efficient doses in patients (3). These important limitations have encouraged extensive exploration of cisplatin combination therapies, of which several are in wide clinical use today. Combination therapies in general are becoming increasingly important within the field of cancer therapy, due to multiple factors such as the potential of re-sensitizing resistant cancers, potentiating the effects of therapy, and/or reducing side effects by facilitating lowered therapeutic doses without compromising the outcome (9). Multiple studies have demonstrated that combination therapies including HSP90 inhibitors can overcome or reverse drug resistance in several cancers due to the wide involvement of HSP90 client proteins in many fundamental aspects of cancer, especially DNA damage response and repair (19, 29, 30). Accordingly, preclinical studies suggest that the HSP90 inhibitor onalespib display radiosensitizing effects, and an ongoing clinical trial is exploring the combination of cisplatin, radiotherapy and onalespib (13, 21, 23). However, little is known about the mechanisms behind the reversal of cisplatin resistance in combination with onalespib and other HSP90 inhibitors.

The four cell lines used in this study were chosen as representatives of types of cancers that are traditionally considered challenging to treat with cisplatin, specifically ovarian and head and neck cancer. Three of the tested cell lines (SKOV3, H314, and A2780CIS) were selected due to their relatively high innate cisplatin resistance (Figure 1), and one was selected as cisplatin sensitive (A2780). As A2780CIS is derived from A2780 a direct comparison of differences in cisplatin sensitivity is possible.

Interestingly, while all cell lines were sensitive to onalespib monotherapy, H314 proved more sensitive than SKOV3, while the A2780 cell lines demonstrated even greater sensitivity. This relationship was consistent in both XTT cytotoxicity and clonogenic survival assays (Figures 1, 3). In this work, however, the combination therapy has been generally found to be more potent than either monotherapy, an effect likely caused by onalespib-mediated inhibition of DNA repair mechanisms activated in response to cisplatin-mediated DNA damage (Figures 2, 3). Combination treatment with 10 μM cisplatin resulted in significantly reduced cell viability for all cell lines at all tested onalespib concentrations as measured by XTT assays (Figure 2). Moreover, combination treatment virtually eliminated H314 and A2780 cell viability and significantly reduced SKOV3 and A2780CIS cell colony formation ability (Figure 3). These findings are in line with previous studies on cisplatin in combination with other HSP90 inhibitors, and are encouraging for the prospect of utilizing cisplatin against types of cancer typically not considered sensitive (30).

Recent studies indicate that the HSP90 inhibitors 17-AAG and AUY922 also can affect cancer cell motility and migration (31–34). To investigate whether these effects translate to onalespib, the effects of cisplatin and/or onalespib on migration and wound healing were investigated in two separate migration assays (Figure 4). Onalespib indeed affected the rate of wound healing while cisplatin did not, thus indicating a reduction in migration and proliferation in both tested cell lines. This reduction was amplified in combination with cisplatin in a dose-dependent manner. Interestingly, H314 cells were unable to migrate in the *trans-*well migration assay, whereas SKOV3 cell migration was impaired in a dose-dependent manner following both cisplatin and onalespib treatment (Table 1). This strongly indicates that the effects on the H314 cells in the wound healing assay were primarily due to reduced proliferation and not impaired migration, which was not the case for the SKOV3 cells.

Interestingly, cisplatin itself inhibits HSP90 by binding to the ATP-binding domain on the C-terminal of HSP90 (2). This raises the question of why HSP90 inhibition prevents or reverses cisplatin resistance when cisplatin itself acts as an HSP90 inhibitor. In light of this, the current findings may seem implausible. Our data clearly demonstrates that the tested HSP90 client proteins are not significantly affected by cisplatin treatment alone, however (Figure 5), whereas expression of HSP90 client proteins EGFR and its downstream target protein AKT were significantly downregulated by onalespib monotherapy at both 50 and 100 nM doses (Figure 5). The high potency of onalespib in terms of downregulation of HSP90 client proteins made it difficult to assess potential combination effects through Western blotting, which has a low dynamic range. There was a trend toward slightly greater downregulation in combination treatments observed for the measured EGFR-expression levels, however, most notably evident in the highest dose combinations. These results indicate that cisplatin-induced DNA damage does not significantly affect HSP90 client protein expression levels. Interestingly, only combination therapy managed to significantly increase the number of apoptotic SKOV3 cells (Figure 6). This finding is consistent with the HSP90 inhibitor geldanamycin demonstrating depletion of essential anti-apoptotic proteins and resulting in greater levels of apoptosis in combination with cisplatin, as demonstrated elsewhere (35). In general, apoptotic cell death is induced by stress, e.g., the withdrawal of stimulating growth factors, hypoxia and DNA damage. The same stimuli induce the expression and accumulation of members of the HSP family, however, including HSP70 and HSP90, which shows that a death stimulus can cause a protective response in the cell (36). HSP90 associates with essential stress-signal- and apoptotic molecules, thereby blocking programmed cell death and promoting survival, proliferation, migration and differentiation, which can be reversed by HSP90 inhibition.

Cisplatin induces DNA intra-strand crosslinks that activate a cascade of DNA damage response (DDR) pathways such as cell cycle arrest, DNA repair and apoptosis (37). The main repair mechanism for cisplatin-induced cross-links is nucleotide excision repair (NER) (38). ERCC1 is a central component of NER and ERCC1 overexpression correlates with cisplatin resistance, indicating its role in the repair of cisplatin-induced DNA damage (39). Excision of cisplatin-induced DNA-adducts through NER can produce DSBs, which are harder to repair for the cell compared to single strand breaks. DNA repair proteins such as ATM, ATR and DNA-PKcs are known client proteins of HSP90, which was also confirmed by Western blotting in this study (Figure 5). Therefore, onalespib-induced HSP90 inhibition may further impair DDR pathways, resulting in increased conversion of single strand breaks to DSBs and a switch from NER to DSB repair mechanisms. The two primary DSB repair mechanisms are homologous recombination (HR) and non-homologous end joining (NHEJ) and the choice of mechanism is strongly connected to cell cycle phases (40). One of the earliest events in the DSB repair is the phosphorylation of H2AX and subsequent phosphorylation of 53BP1 (41, 42). Our studies demonstrate a significant increase in γH2AX and 53BP1 foci and therefore an increase in DSBs of SKOV3 and A2780CIS cells in the combination therapy compared to cisplatin and onalespib monotherapy (Figure 7). This observation proves that the SKOV3 and A2780CIS cells were unable to successfully repair DSBs induced by cisplatin when combined with onalespib, whereas repair in the monotherapy groups was more successful.

## Conclusion

In conclusion, our findings support HSP90 inhibition as a potentially valuable mechanism for enhancing cisplatin efficacy; by increasing the cytotoxic effect, restoring sensitivity in innately resistant cells and possibly preventing development of cisplatin resistance. Further development of this concept has the potential to increase cure rates, prolong survival and increase quality of life for a broad population of patients, and follow-up studies exploring optimal dosing intervals and *in vivo* efficacy are therefore warranted.

## Data Availability Statement

The datasets generated for this study are available on request to the corresponding author.

## Author Contributions

AM contributed to experimental studies with focus on the XTT assays and western blotting, analyzed and interpreted the data, and drafted and revised the manuscript. TM contributed to experimental studies with focus on the western blotting, viability, and migration assays, analyzed and interpreted the data, and drafted and revised the manuscript. MH contributed to experimental studies with focus on the microscopy and flow cytometry assays, analyzed and interpreted the data, and drafted and revised the manuscript. MP contributed to experimental studies with focus on the viability assays, contributed to the data interpretation, and revised the manuscript. DS initiated and designed the study, contributed to data analysis and interpretation, and drafted and revised the manuscript. All authors have read and approved the final manuscript.

## Conflict of Interest

The authors declare that the research was conducted in the absence of any commercial or financial relationships that could be construed as a potential conflict of interest.

## Supplementary Material

FIGURE S1 XTT cell viability normalized to untreated controls of **(A)** SKOV3 and **(B)** H314 cells treated with 0–3000 nM onalespib or the combination of onalespib with 500 nM cisplatin 24 h post treatment. *N* = 3, error bars represent SEM. Dotted lines represent the viability of cisplatin monotherapy at either 500 nM or 10 μM. **(C)** Wound healing/scratch assay. Effect of Cisplatin monotreatment on SKOV3 and H314 cells. **(D)** Representative *trans-*well migration assay images of SKOV3 cells after treatment with onalespib, cisplatin and their combination. The size bar corresponds to 4 μm.

FIGURE S2 Flow cytometric analysis. **(A,B)** Annexin V median fluorescent intensity (MFI) expression (FITC.A+) in SKOV3 cells and **(F,G)** A2780CIS cells after 96 h exposure to 500 nM cisplatin, 100 nM onalespib, or a combination. **(C,D)** Caspase 3/7 median fluorescent intensity (MFI) expression (FITC.A+) in SKOV3 cells and **(F,G)** A2780CIS cells after 96 h exposure to 500 nM cisplatin, 100 nM onalespib, or a combination. Z-VAD-FMK analysis in **(E)** SKOV3 cells and **(J)** A2780CIS cells. *N* = 3, error bars represent SEM. ^∗^*p* < 0.05, ^∗∗^*p* < 0.01, ^∗∗∗^*p* < 0.001, ^****^*p* < 0.0001.

TABLE S1 Average plating efficiency (PE) and standard deviation (SD) of SKOV3, H314, A2780 parental, and A2780 cisplatin resistant cells, *N* > 3.
