# Correction: Overcoming limitations of cisplatin therapy by additional treatment with the HSP90 inhibitor onalespib

**DOI:** 10.3389/fonc.2026.1824012

**Published:** 2026-05-12

**Authors:** Anja Charlotte Lundgren Mortensen, Tabassom Mohajershojai, Mehran Hariri, Marika Pettersson, Diana Spiegelberg

**Affiliations:** 1 Department of Immunology, Genetics and Pathology, Uppsala University, Uppsala, Sweden; 2 Department of Surgical Sciences, Uppsala University, Uppsala, Sweden

**Keywords:** cisplatin, Hsp90 inhibition, drug resistance, synergy, combination treatment, chemo-sensitization, AT13387, CDDP

There was a mistake in **Figure 3A** as published, where the representative image for SKOV3 control was not accurately displayed. The corrected **Figure 3A** appears below.

**Figure 3:**
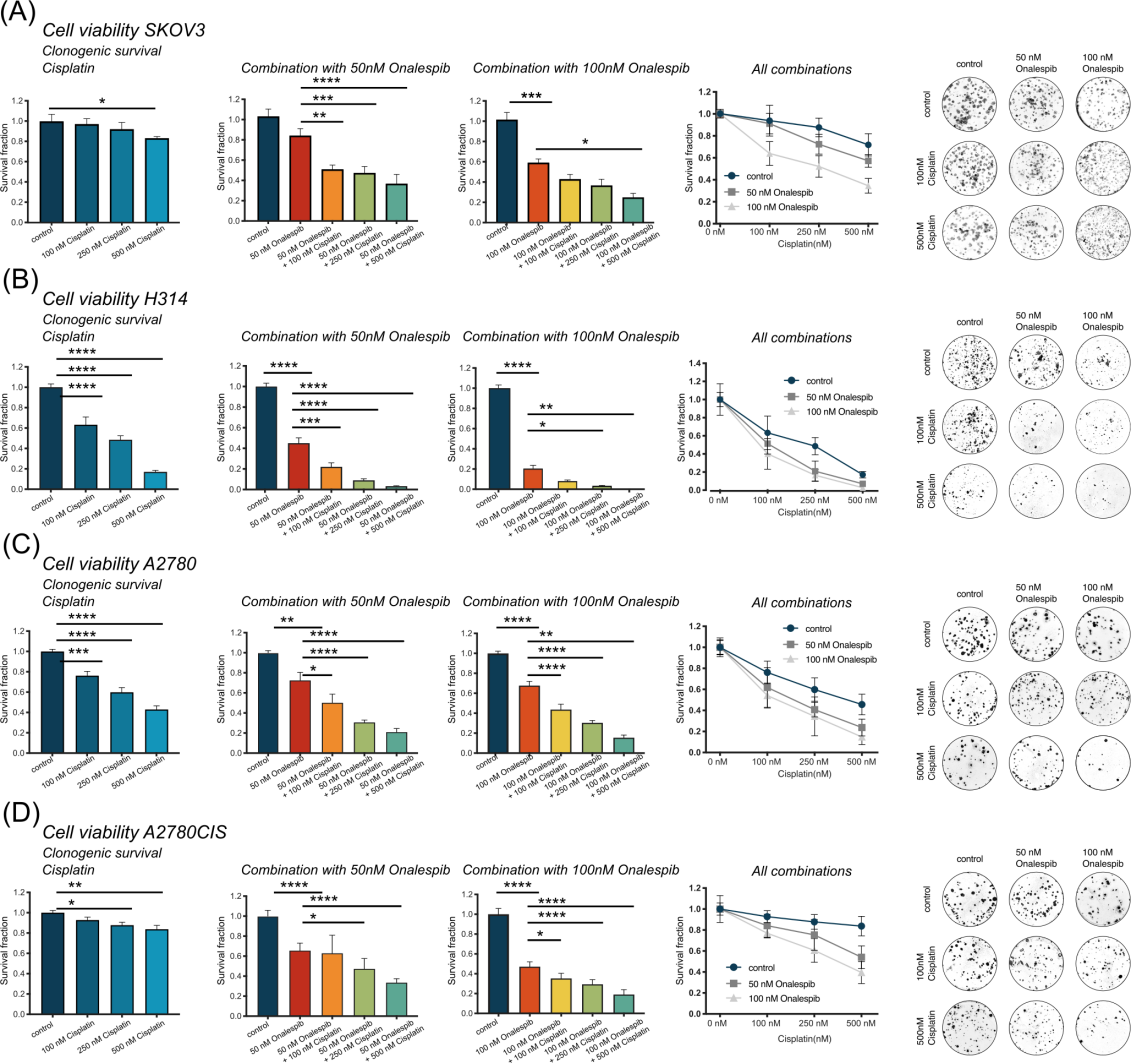
**(A)** Clonogenic survival of SKOV3 cells treated with 0, 100, 250, and 500 nM cisplatin as monotherapy or in combination with 50 or 100 nM onalespib. Note that combination samples are normalized to 0, 50, or 100 nM of onalespib to compensate for the effect on survival by onalespib alone. **(B)** Clonogenic survival of H314 cells treated with 0, 100, 250, and 500 nM cisplatin as monotherapy or in combination with 50 or 100 nM onalespib. Note that combination samples are normalized to 0, 50, or 100 nM of onalespib to compensate for the effect on survival by onalespib alone. **(C)** Clonogenic survival of A2780 cells treated with 0, 100, 250, and 500 nM cisplatin as monotherapy or in combination with 50 or 100 nM onalespib. **(D)** Clonogenic survival of A2780CIS cells treated with 0, 100, 250, and 500 nM cisplatin as monotherapy or in combination with 50 or 100 nM onalespib. Note that combination samples are normalized to 0, 50, or 100 nM of onalespib to compensate for the effect on survival by onalespib alone. N = 3, error bars represent SD. *p < 0.05, **p < 0.01, ***p < 0.001, ****p < 0.0001.

The original version of this article has been updated.

