# Supplementary figures and images for: Overcoming Limitations of Cisplatin Therapy by Additional Treatment With the HSP90 Inhibitor Onalespib
Source: Front Oncol. 2020 Sep 30;10:532285. doi: 10.3389/fonc.2020.532285 (PMC7554556; doi:10.3389/fonc.2020.532285)

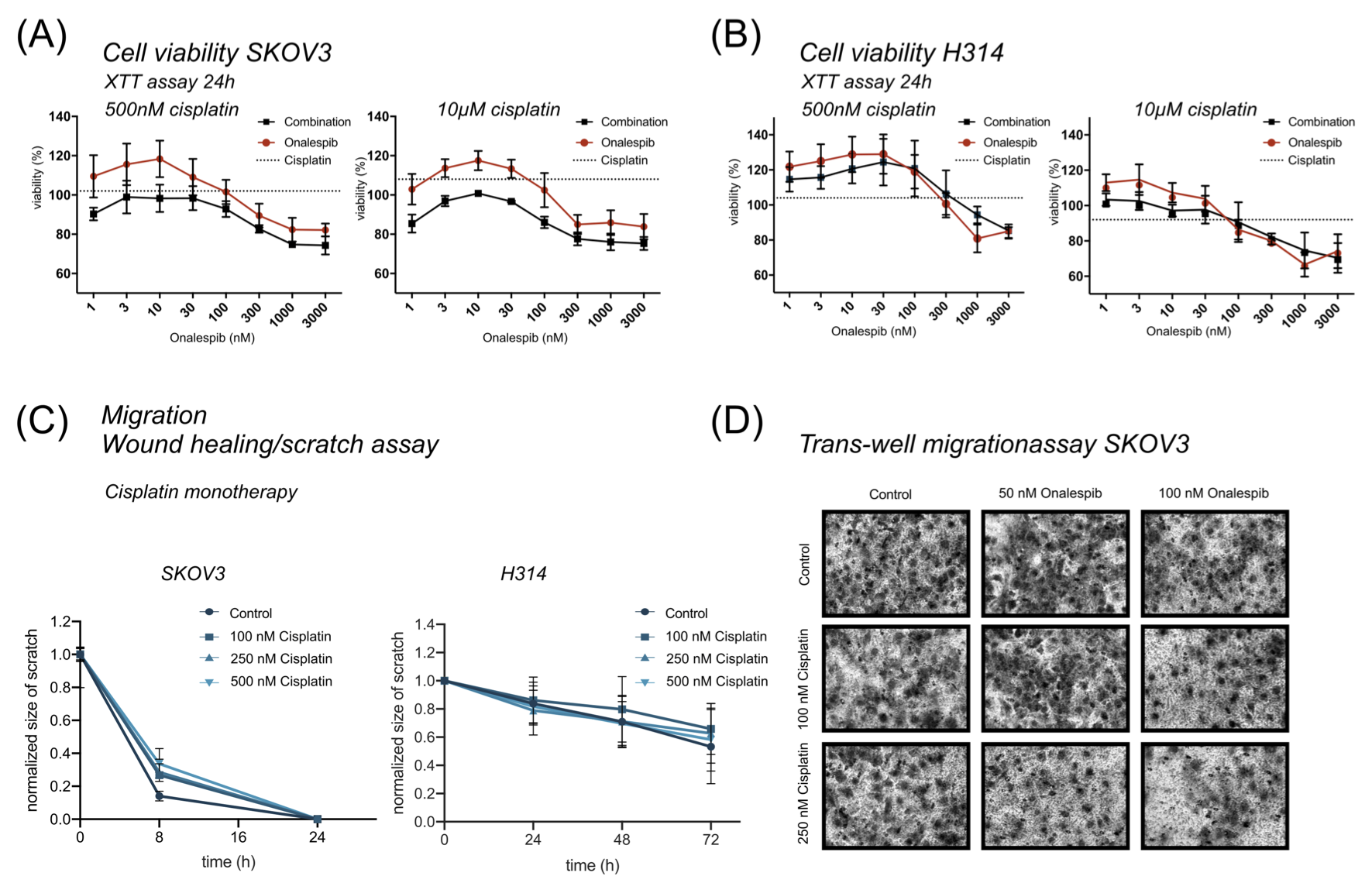

Supplement: FIGURE S1 — XTT cell viability normalized to untreated controls of (A) SKOV3 and (B) H314 cells treated with 0–3000 nM onalespib or the combination of onalespib with 500 nM cisplatin 24 h post treatment. N = 3, error bars represent SEM. Dotted lines represent the viability of cisplatin monotherapy at either 500 nM or 10 μM. (C) Wound healing/scratch assay. Effect of Cisplatin monotreatment on SKOV3 and H314 cells. (D) Representative trans-well migration assay images of SKOV3 cells after treatment with onalespib, cisplatin and their combination. The size bar corresponds to 4 μm. [file Image_1.TIFF]

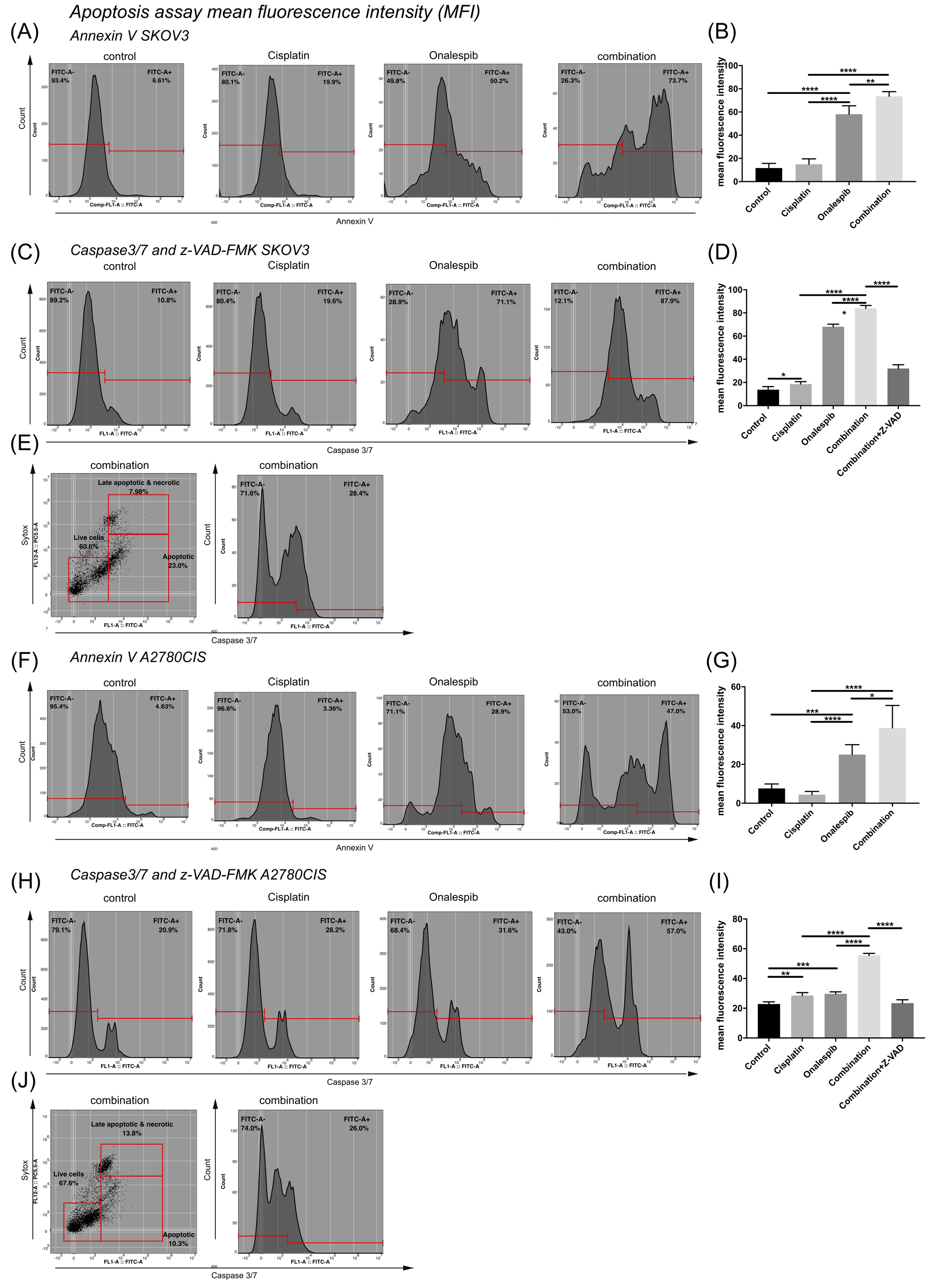

Supplement: FIGURE S2 — Flow cytometric analysis. (A,B) Annexin V median fluorescent intensity (MFI) expression (FITC.A+) in SKOV3 cells and (F,G) A2780CIS cells after 96 h exposure to 500 nM cisplatin, 100 nM onalespib, or a combination. (C,D) Caspase 3/7 median fluorescent intensity (MFI) expression (FITC.A+) in SKOV3 cells and (F,G) A2780CIS cells after 96 h exposure to 500 nM cisplatin, 100 nM onalespib, or a combination. Z-VAD-FMK analysis in (E) SKOV3 cells and (J) A2780CIS cells. N = 3, error bars represent SEM. ∗p < 0.05, ∗∗p < 0.01, ∗∗∗p < 0.001, ****p < 0.0001. [file Image_2.TIFF]
