# Supplementary material for: Overcoming Limitations of Cisplatin Therapy by Additional Treatment With the HSP90 Inhibitor Onalespib
Source: Front Oncol. 2020 Sep 30;10:532285. doi: 10.3389/fonc.2020.532285 (PMC7554556; doi:10.3389/fonc.2020.532285)
Supplement: TABLE S1 — Average plating efficiency (PE) and standard deviation (SD) of SKOV3, H314, A2780 parental, and A2780 cisplatin resistant cells, N > 3. [file Table_1.docx]

Supplementary table 1. Average plating efficiency (PE) and standard deviation (SD) of SKOV3, H314, A2780 parental and A2780 cisplatin resistant cells, N>3.

|  | **SKOV3** | | **H314** | | **A2780** | | **A2780CIS** | |
| --- | --- | --- | --- | --- | --- | --- | --- | --- |
|  | **PE** | **SD** | **PE** | **SD** | **PE** | **SD** | **PE** | **SD** |
| **control** | **0.55** | **0.16** | **0.36** | **0.04** | **0.46** | **0.06** | **0.52** | **0.04** |
| **100 nM cisplatin** | **0.53** | **0.12** | **0.22** | **0.06** | **0.35** | **0.07** | **0.46** | **0.08** |
| **250 nM cisplatin** | **0.51** | **0.13** | **0.17** | **0.02** | **0.27** | **0.05** | **0.47** | **0.04** |
| **500 nM cisplatin** | **0.46** | **0.03** | **0.06** | **0.01** | **0.20** | **0.03** | **0.44** | **0.06** |
| **50 nM Onalespib** | **0.45** | **0.14** | **0.16** | **0.03** | **0.36** | **0.06** | **0.33** | **0.03** |
| **100 nM Onalespib** | **0.32** | **0.06** | **0.07** | **0.03** | **0.34** | **0.04** | **0.24** | **0.03** |
